# Perceptual Awareness Negativity—Does It Reflect Awareness or Attention?

**DOI:** 10.3389/fnhum.2021.742513

**Published:** 2021-10-14

**Authors:** Michał Bola, Łucja Doradzińska

**Affiliations:** Laboratory of Brain Imaging, Nencki Institute of Experimental Biology of Polish Academy of Sciences, Warsaw, Poland

**Keywords:** consciousness, attention, ERP, awareness, visual perception

## Introduction

Identifying the neural correlates of consciousness (NCC) is one of the most important endeavors in neuroscience. A key debate concerns the question whether conscious experience is generated by the early activity of the sensory cortex, or rather by later activity of the supra-modal parietal and frontal cortices. Initial research using the event-related potentials (ERP) technique supported the “late” view, with many studies concluding that the P3b ERP component - a positive wave occurring over centro-parietal regions around 300–500 ms after the stimulus - reflects the mechanism of consciousness (review: Dehaene and Changeux, 2011). However, more recent work reveals, first, that some clearly perceived stimuli do not evoke P3b, meaning this component is not necessary for consciousness; and second, that P3b can be evoked or modulated by subliminal stimuli, which indicates it is not sufficient either. Along with this evidence challenging P3b as NCC, several lines of research have provided strong support for the “early” view. Specifically, a growing body of evidence shows that becoming aware of a stimulus is reliably associated with a negative-going ERP component observed as early as 150–250 ms after the stimulus onset over the modality-specific sensory regions. Such awareness-related negative components have been found in visual, auditory, and somatosensory modalities, and therefore an overarching term Perceptual Awareness Negativity (PAN) has been recently proposed (Dembski et al., 2021).

Recent progress in investigating the mechanisms of perceptual awareness using ERPs has been reviewed by Förster et al. (2020) and Dembski et al. (2021). Both reviews argue that there is enough evidence to falsify P3b as a marker of consciousness, and conclude that PAN is at present the best candidate for NCC. While we generally agree with their view, in this opinion piece we point out that a key challenge faced by the proponents of early NCC is disentangling mechanisms of consciousness from mechanisms of attention. Assuming that PAN reflects the phenomenal aspect of consciousness (Koivisto et al., 2017; Derda et al., 2019), which is by definition orthogonal to attention (Lamme, 2003), dissociating ERP correlates of both processes should be in principle possible. However, we think that conclusive evidence for such a dissociation has not been provided so far and, what is more, a body of data suggests PAN might be in fact closely related to attention.

## ERP Markers of Awareness and Attention

Neural mechanisms of spatial and feature-based attention share many similarities with the postulated early NCC - both are rapid, often automatic, and operate in the sensory cortex *via* feedback connections (Koch and Tsuchiya, 2007; Moore and Zirnsak, 2017). In fact, electrophysiological research has consistently shown that allocating visual attention to a given stimulus is reflected by a more negative component detected at the parieto-occipital electrodes around 200 ms after the stimulus (*selection negativity*, SN; Harter and Aine, 1984), which is particularly prominent contralaterally to the stimulus presentation (*N2 posterior-contralateral*, N2pc; Eimer, 1996). Thus, both visual PAN and attention-related negativity are detected at the same electrodes and in the same time-window, and both are most prominent in the contralateral hemisphere - the only difference is that PAN is defined in relation to activity evoked by unconscious stimuli, while attention-related components in relation to the activity evoked by task-irrelevant stimuli (i.e., presented in irrelevant locations or lacking relevant features). But the fact that these components are defined in relation to a different baseline does not preclude that they are generated by the same source and reflect the same underlying process.

The relation between feature-based attention and early NCC has been investigated by several studies. In the early work of Koivisto et al. PAN was repeatedly observed in response to all aware stimuli - both task-relevant targets and task-irrelevant distractors - irrespective of their status (Koivisto et al., 2005, 2006, 2009; Koivisto and Revonsuo, 2007). These observations suggested that PAN is encapsulated from the influence of feature-based attention and the cognitive aspects of processing, and thus taken as evidence that it represents phenomenal awareness. However, we think these conclusions should be treated with caution as, first, the discussed studies tested small groups of participants by today's standard (between 10 and 12); and second, on a non-significant ANOVA interaction (between stimulus relevance and awareness), whereas such absence of evidence cannot be treated as evidence of no effect (Wagenmakers et al., 2018). More recent studies reported that PAN is indeed evoked by task-irrelevant distractors, in line with results of Koivisto and colleagues; but found also that task-relevant stimuli evoke PAN of significantly greater amplitude than the task-irrelevant ones (Pitts et al., 2014; Shafto and Pitts, 2015; Schelonka et al., 2017). This strongly suggests that PAN is influenced by the task-related factors, and thus challenges earlier conclusions of Koivisto et al. (2005, 2006, 2009). Yet, it is worth emphasizing that some early studies observed differences in the spatio-temporal profiles of awareness- and attention-related early ERP effects (Koivisto et al., 2005; Koivisto and Revonsuo, 2007). Thus, in such a scenario greater PAN to task-relevant stimuli will reflect an overlap of two independent components [as discussed by Pitts et al. (2014)]. Therefore, the putative functional dissociation of awareness and feature-based attention should be the focus of future studies.

In another study Koivisto et al. (2009) investigated how PAN interacts with the top-down spatial attention. Stimuli were displayed either in the attended or in the unattended visual field, and participants were either aware or unaware of their presentation. Quite strikingly, clearly visible stimuli did not evoke PAN when displayed in the unattended visual field. This result is in line with an earlier study, in which PAN was greatly reduced in a passive viewing condition, which did not require focused attention (Koivisto and Revonsuo, 2008). While these findings might indicate that attention is a prerequisite of consciousness, as argued by Koivisto et al. (2009), such an interpretation will be difficult to reconcile with the assumption that PAN reflects an elementary phenomenal experience (e.g., Koivisto et al., 2017). Because implications of the Koivisto et al. (2009) study are of crucial importance, we argue that the relation between spatial attention and early NCC should be further investigated.

While so far we have discussed to what extent PAN is influenced by the top-down feature-based or spatial attention, another question is whether the same type of activity can be evoked by unconscious stimuli that are salient or task-relevant. This question is of particular importance, since the attention-related early negative components can be evoked by unconscious stimuli (Koivisto and Revonsuo, 2007; Travis et al., 2019; Bola et al., 2021). But finding that PAN can be evoked or modulated unconsciously would falsify this component as an NCC (similarly to P3b; Silverstein et al., 2015; Doradzińska et al., 2020) and might suggest it is more closely related to attentional prioritization. It is thus important to point out that Koivisto and Grassini (2016) found that unaware but correct trials - those in which participants reported not seeing a stimulus but provided a correct response in a forced-choice task - were characterized by more negative amplitude in the PAN spatio-temporal window, in comparison to unaware and incorrect trials. They interpreted this effect as reflecting residual awareness in the “unaware” but correct trials, not reported by some participants due to a conservative bias. But an equally likely interpretation is that PAN might reflect greater involvement of attention and more extensive processing in some of the unaware trials, and thus that it can be evoked outside of awareness. Of note, a similar effect has been observed by Eklund and Wiens (2018; see their Figure 2), but it was not analyzed statistically^1^. We understand that the main argument against our reasoning might be as follows: *PAN is defined as a difference between aware and unaware presentations, and thus there is no such thing as an “unconscious PAN”*; but we again emphasize, that such a definition does not preclude the very same type of activity that constitutes PAN to be related to other mechanisms and thus be observed in other comparisons. Testing this possibility will be crucial for establishing PAN as NCC. Therefore, future research should identify the sources or spatio-temporal filters representing PAN according to the classic definition (i.e., comparison between aware and unaware trials) and then apply them to compare activity observed in the unconscious conditions, for instance evoked by salient and neutral subliminal stimuli. Such a research strategy will allow determining whether the “PAN-like” negativity seen in the unconscious trials reflects the same process as the classically defined PAN.

Importantly, while results discussed so far cast doubt on the function of PAN as a marker of awareness, the exact function of attention-related components is also a matter of debate. For instance, while N2pc is classically interpreted as reflecting attention shifts, robust N2pc has been repeatedly observed without an accompanying behavioral effect of attention (Kappenman et al., 2014). Further, N2pc is unaffected by attentional cueing - its amplitude is the same in response to stimuli presented in the cued and uncued locations (Brisson and Jolicoeur, 2008; Kiss et al., 2008). Both findings suggest a possibility that N2pc does not reflect attention shifts *per se*, but rather a different, co-occurring process. In line with this hypothesis, recent data indicates that N2pc might rather represent engagement of attention in binding and integration of features (Zivony et al., 2018). Indeed, in a change blindness study PAN was related to a mere detection that something has changed, while N2pc occurred only when subjects were able to identify a change (Busch et al., 2010). If N2pc is indeed a marker of binding and integration of information then many electrophysiological findings interpreted as reflecting mechanisms of attention might, in some way, be more closely related to the mechanism of consciousness (Mudrik et al., 2014).

Finally, while in this commentary we focus on discussing attention and consciousness in the visual domain, one of the key arguments of Dembski et al. (2021) is that PAN is common across modalities - visual, auditory, and somatosensory - and can be observed in respective sensory cortices. Here we want to emphasize two points. First, because the attention-related negativity occurs also in the auditory domain (Alho et al., 1987; Gamble and Luck, 2011; Luck and Kappenman, 2012), the discussed relation between neural correlates of attention and consciousness should be investigated also in the auditory, and in other modalities. Second, the modality-specific neural mechanisms are expected for attention, the role of which is to prioritize stimuli from a particular modality. But in case NCC a mechanism integrating these unimodal experiences into a supra-modal, coherent, and unified conscious experience must be proposed (Mudrik et al., 2014). Because such a mechanism has so far not been proposed or investigated in the context of PAN, this might be considered a theoretical argument against early NCC in general, and PAN in particular.

## Conclusions

Förster et al. (2020) and Dembski et al. (2021) provided a detailed overview of the recent electrophysiological research on NCC. Both reviews concluded that a robust body of evidence supports the early Perceptual Awareness Negativity (PAN) as an ERP correlate of consciousness. While we agree that PAN is at present the most promising candidate for NCC, in this opinion article we have discussed evidence indicating that PAN might be closely related to mechanisms of attention. Relevant data is at present scarce, but there is evidence indacting PAN is not necessary for consciousness (Koivisto et al., 2009), and that it might not be sufficient either (Koivisto and Grassini, 2016; Eklund and Wiens, 2018). These effects have so far not been discussed critically but, if further confirmed, they might challenge the dominant interpretation of PAN as a correlate of phenomenal awareness. Therefore, an important message of the present article is that a falsification-based approach should be more often embraced when investigating PAN, similarly as has been done in case of P3b.

In conclusion, while proponents of the “early” view have falsified the P3b component as an NCC by showing that it reflects mainly task-related cognitive processing, they now face a challenge of proving that “A” in PAN indeed stands for awareness and not attention.

## Author Contributions

MB and ŁD discussed the presented ideas. MB drafted the initial version. All authors revised the manuscript.

## Funding

This work was supported by National Science Center Poland (Grants Nos.: 2018/29/B/HS6/02152 and 2019/33/B/HS6/02233).

## Conflict of Interest

The authors declare that the research was conducted in the absence of any commercial or financial relationships that could be construed as a potential conflict of interest.

## Publisher's Note

All claims expressed in this article are solely those of the authors and do not necessarily represent those of their affiliated organizations, or those of the publisher, the editors and the reviewers. Any product that may be evaluated in this article, or claim that may be made by its manufacturer, is not guaranteed or endorsed by the publisher.

## Footnotes

^1^Interestingly, PAN differed between correct and incorrect unaware trials in studies of Koivisto and Grassini (2016) and Eklund and Wiens (2018), in which stimuli were presented centrally (always in the same location); but not in the studies of Lamy et al. (2009) and Salti et al. (2012), in which stimuli were presented in one of the uncued peripheral locations. Our putative interpretation is that in the latter studies the top-down spatial attention was not sufficiently involved and therefore PAN was not observed [in line with results of the Koivisto et al. (2009) study, discussed in the previous paragraph].
